# Deferral of scheduled transcatheter heart valve interventions strongly increases the risk of congestive heart failure

**DOI:** 10.1038/s41598-025-16742-7

**Published:** 2025-09-01

**Authors:** Stefanie Andreß, Dominik Felbel, Sascha d’Almeida, Dominik Buckert, Wolfgang Rottbauer, Armin Imhof, Tilman Stephan

**Affiliations:** Department of Internal Medicine II, University Hospital Ulm, Albert-Einstein-Allee 23 , 89081 Ulm, Germany

**Keywords:** Waiting time, Covid-19 pandemic, Epidemiology, Heart failure, Heart valve interventions, Interventional cardiology, Risk factors, Epidemiology, Preventive medicine

## Abstract

Deferral of non-emergency cardiac interventions is associated with worse clinical outcomes, even post-procedurally. Affected patients show signs of congestive heart failure (CHF) after the waiting time. To identify predictors of CHF with clinical progress during prolonged waiting time, and assess the impact of CHF on the actual intervention date and of the identified baseline predictors in case of deferral on subsequent outcomes. Consecutive patients whose non-emergency cardiac intervention was postponed during the first Covid-19 related lockdown between March 19th and April 30th, 2020 were included (n = 178). Binary logistic regression analysis was performed to identify predictors of clinically progredient CHF, indicated by an NT-proBNP level of > 900 pg/ml on the actual intervention date in combination with worsening of dyspnea as assessed by NYHA class, emergency heart failure hospitalization, or declining left ventricular ejection fraction (LVEF) during the waiting time. Clinical outcomes were compared to a seasonal control group undergoing such interventions in 2019 as scheduled (n = 214). 89 of 178 deferred patients (50.0%) had an NT-proBNP level of > 900 pg/ml in combination with clinical symptoms indicating CHF on the actual intervention date. Thereof, repeated data measurement was available for 72 patients, of whom 54 (75%) experienced new-onset or worsening of CHF, while 18 (25%) had stable, pre-existing CHF. Planned transcatheter heart valve intervention was the only independent predictor of CHF with clinical progress (OR 34.632, 95%-CI 3.337–359.404, *p* = 0.003). Risk was even higher in patients with planned mitral or tricuspid edge-to-edge-repair (M/T-TEER) than in those with transcatheter aortic valve replacement (TAVR) (82.4% vs. 40.0%; *p* = 0.035 after Bonferroni correction). During the post-procedural 36-month follow-up, rates of emergency hospitalization or death were significantly higher in patients with CHF and time-to event was shorter compared to those without (57.3% vs. 14.0%, *p* < 0.001; HR 6.432, 95%-CI 3.476–11.868; log rank *p* < 0.001). Higher event rates and shorter time-to-event in deferred compared to regularly treated patients after the originally planned intervention date were observed only in those with the predictor scheduled heart valve intervention (83.7% vs. 40.4%, *p* < 0.001; HR 4.37, 95%-CI 2.50–7.64, log rank *p* < 0.001). Deferral of planned transcatheter heart valve intervention, TAVR and especially M/T-TEER, leads to a highly increased risk of clinically progredient CHF during prolonged waiting time and worse clinical outcomes. Therefore, these procedures should be prioritized and postponement should be avoided.

**Supplementary Information:**

The online version contains supplementary material available at 10.1038/s41598-025-16742-7.

## Introduction

Congestive heart failure (CHF) is one of the leading causes of hospitalization and death worldwide^1^. According to the universal definition CHF is a clinical syndrome with symptoms and/or signs caused by a structural and/or functional abnormality and corroborated by elevated natriuretic peptide levels and/or objective evidence of pulmonary or systemic congestion^2^. In developed countries, the prevalence is estimated to be 1–4% and is projected to rise dramatically due to ageing populations^3^. With over 64 million people affected worldwide resulting in high morbidity and mortality rates, CHF already represents a great and increasing burden to healthcare system^4–6^. In a large meta-analysis including 1.5 million patients with chronic heart failure survival rates were 95.7% at 30 days, 86.5% at one year, 72.6% at two years, 56.7% at five years and only 34.9% at ten years^7^. In patients with acute heart failure, survival rates are even lower with 55–65% at one year^8,9^. Thus, it is essential to prevent the development of CHF in advance by adequate and timely therapy of cardiac disease^10^. However, during the Covid-19 pandemic, hospitals were forced to postpone non-emergency interventions to increase capacity for SARS-CoV2-infected patients^11^. Previous studies have demonstrated that deferral of cardiovascular procedures was associated with poor clinical outcomes^10,12^. During the waiting time affected patients developed increasing NT-proBNP levels indicating cardiac impairment^10^. As already shown, the risk of adverse outcomes is strongly predicted by elevated natriuretic peptides even in patients without heart failure^13^. Even post-procedure these high NT-proBNP values persisted suggesting ongoing cardiac damage^10^. Therefore, the present study aimed to identify patients at risk for developing clinically progredient CHF during prolonged waiting time in advance to better assess urgency and provide them timely treatment in the future.

## Methods

### Study design

This retrospective observational case control study included all consecutive patients, whose non-emergency appointments had been deferred at Ulm University Heart Center, Germany, during the first Covid-19-related lockdown between March 19th, 2020 and April 30th 2020 (study group). Patients eligible for inclusion were scheduled for elective (1) transcatheter heart valve intervention, (2) cardiac catheterization due to suspected or known significant coronary artery disease, (3) electrophysiological intervention, or (4) device implantation (implantable cardioverter defibrillator or permanent pacemaker). All patients of the study group were classified as ‘non-emergency’ or ‘elective’ and therefore considered as deferrable according to the current recommendations of the European Society of Cardiology (ESC) at that time^11^. Patients who did not subsequently undergo the intended intervention later were excluded from this study. Consecutive non-emergency cardiac patients who received their planned intervention as scheduled in the corresponding period of the previous year (March 19th to April 30th 2019) served as control group. All patients gave their informed consent to participate in the study. The study conforms to the guidelines of the Declaration of Helsinki, adheres to the STROBE statement and was approved by the local ethics committee (ethics application 252/20).

### Data collection, follow-up and laboratory procedures

Demographic, clinical and laboratory data at baseline, on the date of the actual performed intervention and at follow-up as well as outcome data were extracted from our patient management system. Missing data were supplemented by telephone interviews. Outpatient clinic visits including clinical assessment and focused cardiovascular examinations such as 12-lead electrocardiogram (ECG), transthoracic echocardiography and laboratory blood tests were routinely carried out one, two and six months after the procedure and thereafter every six months, whenever possible. Symptoms were classified according to the scales of NYHA (New York Heart Association), EHRA (European Heart Rhythm Association) and CCS (Canadian Cardiovascular Society). Left ventricular systolic function (LVSF or left ventricular ejection fraction, LVEF) was measured either by echocardiography (EPIQ 7, Koninklijke Philips N.V., Eindhoven, Netherlands) or ventriculography during cardiac catheterization and categorized as normal, mildly impaired, moderately impaired, or severely impaired, according to guideline specific recommendations^14^. Highly sensitive cardiac troponin T (cTnT), NT-proBNP, and creatinine levels were measured from the blood samples (ElectroChemiLumineszenz ImmunoAssay “ECLIA” Roche, Cobas 8000, Modul e801 and e601). All appointments and data collected were part of our clinical routine.

The primary focus of our study was to identify baseline parameters on the originally planned intervention date that predict CHF with clinical progress during the waiting time. Further investigations evaluated the implications of presence of CHF on the actual intervention date, and of the presence of baseline risk factors for developing CHF with clinical progress during the waiting time in the study and control group on the originally planned intervention date. The primary endpoint of the latter investigations was the rate of emergency hospitalization or death, along with the time to event. Secondary outcomes comprised clinical signs of heart failure measured by NYHA class, plasma cTnT and creatinine levels, LVEF, hospitalization data such as length of stay and need for performance of the planned intervention in the context of an emergency hospitalization.

### Statistical analysis

Continuous variables were presented as mean ± standard deviation or median along with interquartile range (IQR) as appropriate. The Kolmogorov–Smirnov-test was used to assess normal distribution of continuous parameters. If a metric variable was not normally distributed at any time of measurement, all values were presented as median together with the IQR. Categorial variables were described as number and percentage. Student’s t-test, Mann–Whitney U-Test or Chi^2^ test were performed to compare variables between groups where appropriate. To account for multiple comparisons in the subgroup analyses, *p* values were adjusted using the Bonferroni correction.

Binary logistic regression analysis was performed to identify predictors of CHF with clinical progress during the waiting time. The target variable of the regression analysis, CHF with clinical progress during the waiting time, was defined as elevated NT-proBNP levels above the cut-point of 900 pg/ml, in combination with new-onset or worsening of CHF symptoms. This included patients who were initially CHF-free and experienced new-onset, as well as patients who initially had CHF and experienced CHF-worsening. NT-proBNP levels > 900 pg/ml on the actual intervention date were used as the primary identifier of CHF. In case of additional presence of symptoms, the diagnosis of CHF was made. Patients diagnosed with CHF were grouped according to the course of clinical symptoms into patients with pre-existing, stable CHF, and those with new-onset or worsening of CHF. Clinical symptoms included NYHA class, heart failure emergency hospitalization, and reduced LVEF. These CHF symptoms were assessed on the originally planned intervention date and the actual, deferred intervention date, which marked the beginning and the end of the waiting period. Progressive CHF was diagnosed if at least one of these criteria worsened between these dates. All baseline parameters assessed were included in the binary logistic regression analysis without any exclusion and regardless of their relation in the comparison of patients with and without CHF on the later, actual intervention date. All baseline parameters included in the regression analysis for CHF predictors were collected upon the originally planned treatment date. First, univariate logistic regression analysis was performed for all baseline parameters. Pearson correlation was used to identify and exclude predictors with high collinearity (|r|> 0.7). Parameters with a *p* value < 0.05 in the univariate analysis were included in the multivariate model. The strength of the association was shown as odds ratio (OR) with 95% confidence interval (CI). Receiver operating characteristic (ROC) analysis was performed to assess the performance of the model by estimating sensitivity, specificity and area under the curve (AUC).

Time-to-event analysis was performed for the following analyses: 1. CHF vs. no CHF (as classified by an NT-proBNP level of > 900 pg/ml on the actual intervention date in combination with clinical symptoms or an NT-proBNP level of <  = 900 pg/ml/no symptoms) and 2. presence versus absence of the identified risk factor for CHF with clinical progress during the waiting time, each for the study and control group; with the outcome of both being time to emergency-hospitalization or death. The presence of CHF with clinical progress during the waiting time was assessed on the date of the actual intervention as previously described. As a baseline characteristic, the presence of the risk factor was assessed on the originally planned intervention date, which also determined group membership (study or control group). The time at which these variables were assessed, namely the actual intervention date for CHF status, and the originally planned intervention date for risk factor and group membership, was the starting point of the respective time-to-event analysis. The endpoint was the time to the first event of emergency hospitalization or death. Patients were followed up until they reached the endpoint, were lost to follow-up, or 36 months after the intervention performed. The Kaplan–Meier estimator was used to assess the time to the first event. Right-censoring was performed for patients who were lost to follow-up. Comparison of groups was performed using the Cox proportional hazard model, with results reported as hazard ratios (HR) and 95%-CI.

Parameters with a *p* value < 0.05 were considered statistically significant. Statistical analysis was performed using SPSS Statistics 29 software (Version 2022, IBM, Armonk, NY, USA). Due to the explorative nature of this study, all results of statistical tests have to be interpreted as generating hypothesis, and especially the subgroup analyses are of exploratory nature.

## Results

### Study population

Non-emergency cardiac interventions of 193 consecutive patients were deferred at Ulm University Heart Center, Germany, between March 19th, and April 30th 2020 (study group). 15 patients were excluded because their planned intervention was not performed by the end of the follow-up period. Of the remaining 178 patients, 74 patients (41.6%) underwent cardiac catheterization, 49 patients (27.5%) transcatheter heart valve intervention, 47 patients (26.4%) electrophysiological procedure and 8 patients (4.5%) device implantation. The planned intervention was deferred by a median of 23 (19, 38) days. During the corresponding seasonal period of the previous year 2019 (March 19th–April 30th 2019) 216 patients were scheduled for a non-emergency cardiac intervention at our tertiary care center, of whom 214 patients received their procedure as planned (control group). Distribution of the interventions did not differ significantly between the groups (*p* = 0.397). In the control group 93 patients (43.5%) underwent cardiac catheterization, 47 patients (22.0%) transcatheter heart valve intervention, 57 patients (26.6%) electrophysiological procedures and 17 patients (7.9%) device implantation.

Baseline data on the originally planned intervention date of both groups are displayed in Table 1. In both cohorts, the median age was slightly above 70 years (study group 72.31 ± 11.18 vs. control group 70.29 ± 13.19 years; *p* = 0.105) and most patients were male (study group 59% vs. control group 66%; *p* = 0.142). Patients of the study group suffered more frequently from known coronary artery disease at baseline (76.7% vs. 65.4%, *p* = 0.026) and had a significantly higher heart rate at admission (73 (64, 85) vs. 68 (60, 80) beats per minute (bpm), *p* = 0.014). Furthermore, study group patients had higher cTnT levels at baseline (22.0 (10.75, 36.5) vs. 15.5 (9, 28) ng/l, *p* = 0.030). In the control group, significantly more patients had a positive family history for cardiovascular disease (26.6% vs. 13.8%, *p* = 0.004). Moreover, EHRA class was significantly higher in the control group (1.4 ± 0.8 vs. 1.4 ± 0.6, *p* = 0.006). Beyond that, no significant differences were observed in patients` baseline characteristics.

**Table 1:** Baseline characteristics on the originally planned intervention date: study group (deferred patients) and control group (regularly treated patients).

	Study group	Control group	*p* value
	n = 178	n = 214	
Age (years)	72.31 ± 11.18	70.29 ± 13.19	0.105
Male sex (%)	105 (59.0)	142 (66.4)	0.142
Height (cm)	172.50 ± 9.45	171.27 ± 9.29	0.373
Weight (kg)	86.14 ± 19.07	83.79 ± 17.88	0.385
Heart rate (bpm)	73 (64, 85)	68 (60, 80)	**0.014**
Blood pressure systolic (mmHg)	129.69 ± 19.82	129.20 ± 20.20	0.921
Blood pressure diastolic (mmHg)	75.94 ± 14.40	78.92 ± 12.74	0.390
Arterial hypertension (%)	114 (78.1)	171 (79.9)	0.693
Dyslipidemia (%)	91 (62.8)	154 (72.0)	0.083
Diabetes mellitus (%)	45 (31.0)	50 (23.4)	0.114
Familiy history (%)	20 (13.8)	57 (26.6)	**0.004**
Smoker (%)	52 (35.9)	80 (37.4)	0.824
Obesity (%)	32 (22.1)	54 (25.2)	0.530
History of TIA/stroke (%)	17 (11.7)	17 (7.9)	0.271
COPD (%)	13 (9.0)	13 (6.1)	0.407
OSAS (%)	10 (6.8)	11 (5.1)	0.648
Known CKD (%)	28 (19.2)	36 (16.8)	0.577
Known CAD (%)	112 (76.7)	140 (65.4)	**0.026**
Known cardiac arrhythmia (%)	89 (61.0)	121 (56.5)	0.405
Planned valve intervention (%)	49 (27.5)	47 (22.0)	0.238
Planned rhythmological intervention (%)	55 (30.9)	74 (35)	0.452
Electrophysiological intervention (%)	47 (26.4)	57 (26.6%)	0.440
Device implantation (%)	8 (4.5)	17 (7.9%)	0.112
Planned cardiac catheterization (%)	74 (41.6)	93 (43.5)	0.758
NYHA class			
NYHA class average	1.9 ± 0.8	1.9 ± 0.8	0.402
NYHA I	39 (38.2)	71 (33.6)	
NYHA II	36 (35.3)	86 (40.8)	
NYHA III	27 (26.5)	50 (23.7)	
NYHA IV	0 (0)	4 (19)	
EHRA class	1.4 ± 0.6	1.4 ± 0.8	**0.006**
CCS class	0.7 ± 1.1	0.8 ± 1.2	0.130
cTnT (ng/l)	22.0 (10.75, 36.5)	15.5 (9, 28)	**0.030**
NT-proBNP (pg/ml)	866 (229, 1963)	538.5 (157.75, 1569.25)	0.995
Creatinin (µmol/l)	104.71 ± 53.36	105.80 ± 91.18	0.922
LVEF grade	2.3 ± 1.1	2.1 ± 1.2	0.108
LVEF normal	32 (31.4)	82 (42.3)	
LVEF mildly impaired	26 (25.5)	46 (23.7)	
LVEF moderately impaired	21 (20.6)	22 (11.3)	
LVEF severely impaired	23 (22.5)	44 (22.7)	
Waiting time (days)	23 (19, 38)	0 (0, 0)	** < 0.001**

bpm, beats per minute; TIA, transient ischemic attack; COPD, chronic pulmonary obstructive disease; OSAS, obstructive sleep apnea syndrome; CKD, chronic kidney disease; CAD, coronary artery disease; NYHA, New York Heart Association; EHRA, European Heart Rhythm Association; CCS, Canadian Cardiovascular Society; cTnT, cardiac Troponin T; LVEF, left ventricular ejection fraction (grades: 1 normal, 2 mildly impaired, 3 moderately impaired), 4 severely impaired); significant differences are presented in bold.

### Predictors of CHF with clinical progress during prolonged waiting time

**Fig. 1:**
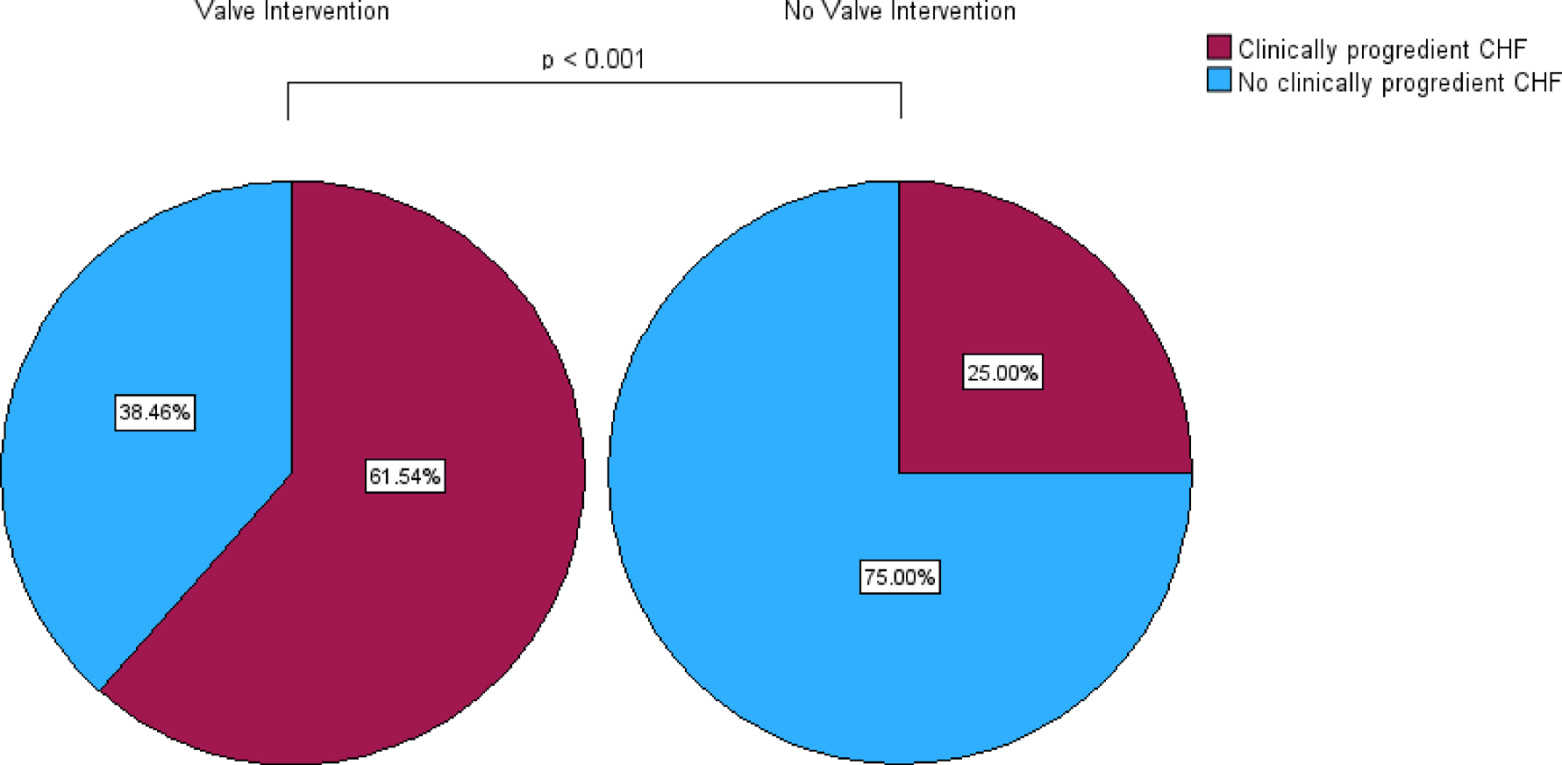
Proportion of deferred patients (study group) with and without clinically progredient congestive heart failure (CHF, NT-proBNP > 900 pg/ml in combination with worsening of clinical symptoms) during the waiting time in the groups with and without planned heart valve intervention.

Univariate analysis showed that planned transcatheter heart valve intervention (*p <*0.001), older age (*p <*0.001), severely reduced LVEF (*p* = 0.003), known cardiac arrhythmia (*p* = 0.010), cTnT levels (*p* = 0.030), known chronic kidney disease (*p* = 0.032), and planned cardiac catheterization (*p* = 0.041) were associated with CHF with clinical progress during the waiting time (NT-proBNP level > 900 pg/ml in combination with worsening of clinical symptoms, including dyspnea, emergency heart failure hospitalization, and LVEF) (supplementary table 1). After exclusion of variables with high collinearity (The corresponding Pearson correlation analysis is shown in supplementary table 2), multivariate logistic regression analysis of all significantly tested variables showed that only planned heart valve intervention was independently associated with CHF with clinical progress (OR 34.632, 95%-CI 3.337–359.404; *p <*0.001) (Table 2). ROC-analysis yielded an AUC of 0.657 (95%-CI 0.576–0.738), a sensitivity of 44.4% and a specificity of 85.7%. Of the patients for whom repeated data assessment was available, 24 of 39 (61.5%) with planned transcatheter heart valve intervention had clinically progredient CHF during the waiting time, in contrast to only 30 of 120 (75.0%) with other cardiac procedures (*p <*0.001) (Fig. 1).

**Table 2:** Multivariate binary regression analysis for baseline characteristics on the originally planned intervention date for congestive heart failure (NT-proBNP level > 900 pg/ml in combination with clinical symptoms) with clinical progress during the waiting time (including univariate predictors *p *< 0.05).

	Odds ratio	95%-Confidence interval	*p* value
Valve intervention	34.632	3.337–359.404	**0.003**
Age	0.983	0.915–1.056	0.636
LVEF	2.568	0.544–12.127	0.234
Known cardiac arrhythmia	3.979	0.867–18.271	0.076
cTnT	1.025	0.993–1.059	0.133
Known CKD	1.173	0.206–6.678	0.858
Planned cardiac catheterization	2.808	0.597–13.213	0.191

LVEF, left ventricular ejection fraction; CKD, chronic kidney disease; significant differences are presented in bold.

### CHF on the actual intervention date

On the actual intervention date, 89 of 178 deferred patients (50.0%) had a plasma NT-proBNP level > 900 pg/ml, indicating CHF (control group with regular treatment time 85 of 214 patients (39.7%), (*p* = 0.078)). All patients with NT-proBNP levels > 900 pg/ml on the actual intervention date additionally had symptoms and thus were diagnosed with CHF. Based on the presence of CHF after the prolonged waiting time, deferred patients were divided into two groups. Baseline data of the patients with and without CHF, assessed on the actual intervention date, are shown in Table 3. Patients with an NT-proBNP level > 900 pg/ml were of older age (77.0 ± 8.8 years vs. 67.6 ± 11.4 years; *p <*0.001) and suffered more often from chronic kidney disease (26.4% vs. 12.3%; *p* = 0.033). Moreover, they were significantly more frequently scheduled for transcatheter heart valve intervention (43.8% vs. 11.4%; *p <*0.001). In contrast, patients with an NT-proBNP level of ≤ 900 pg/ml were more likely to be smoker (44.4% vs. 27.8%; *p* = 0.038) and were more often planned for cardiac catheterization (52.3% vs. 30.3%; *p* = 0.003). All other baseline characteristics were similarly distributed in both populations. The waiting time also did not differ between patients with and without CHF (22 (19, 36) vs. 25 (18, 40) days; *p* = 0.335).

**Table 3:** Baseline characteristics of deferred patients (study group) with and without congestive heart failure (NT-proBNP level > 900 pg/ml in combination with clinical symptoms) on the actual intervention date.

	NT-proBNP > 900 pg/ml + symptoms	NT-proBNP < = 900 pg/ml	*p* value
	n = 89	n = 89	
Age (years)	77.04 ± 8.83	67.60 ± 11.38	** < 0.001**
Male sex (%)	48 (53.9)	56 (63.6)	0.192
Height (cm)	173.79 ± 8.63	171.46 ± 10.20	0.339
Weight (kg)	83.32 ± 17.84	87.34 ± 19.31	0.399
Heart rate (bpm)	75.43 ± 14.89	75.48 ± 18.15	0.989
Blood pressure systolic (mmHg)	125.95 ± 22.20	130.50 ± 17.46	0.309
Blood pressure diastolic (mmHg)	74.05 ± 16.85	76.93 ± 12.32	0.488
Arterial hypertension (%)	60 (83.3)	53 (72.6)	0.121
Dyslipidemia (%)	40 (55.6)	50 (69.4)	0.086
Diabetes mellitus (%)	25 (34.7)	20 (27.8)	0.372
Familiy history (%)	8 (11.1)	12 (16.7)	0.339
Smoker (%)	20 (27.8)	32 (44.4)	**0.038**
Obesity (%)	15 (20.8)	16 (22.2)	0.841
History of TIA/stroke (%)	11 (15.3)	5 (6.9)	0.113
COPD (%)	8 (11.1)	5 (6.9)	0.387
OSAS (%)	5 (6.9)	5 (6.8)	0.982
Known CKD (%)	19 (26.4)	9 (12.3)	**0.033**
Known CAD (%)	56 (77.8)	55 (75.3)	0.730
Known cardiac arrhythmia (%)	104 (68.4)	102 (51.3)	0.405
Planned valve intervention (%)	39 (43.8)	10 (11.4)	** < 0.001**
Planned rhythmological intervention (%)	23 (25.8)	32 (36.4)	0.132
Electrophysiological intervention (%)	28 (16.1)	46 (22.0)	0.141
Device implantation (%)	15 (8.6)	11 (5.3)	0.204
Planned cardiac catheterization (%)	27 (30.3)	46 (52.3)	**0.003**
Waiting time (days)	22 (19, 36)	25 (18, 40)	0.335

bpm, beats per minute; TIA, transient ischemic attack; COPD, chronic pulmonary obstructive disease; OSAS, obstructive sleep apnea syndrome; CKD, chronic kidney disease; CAD, coronary artery disease; significant differences are presented in bold.

### Clinical impact of the presence of CHF on the actual intervention date

**Fig. 2:**
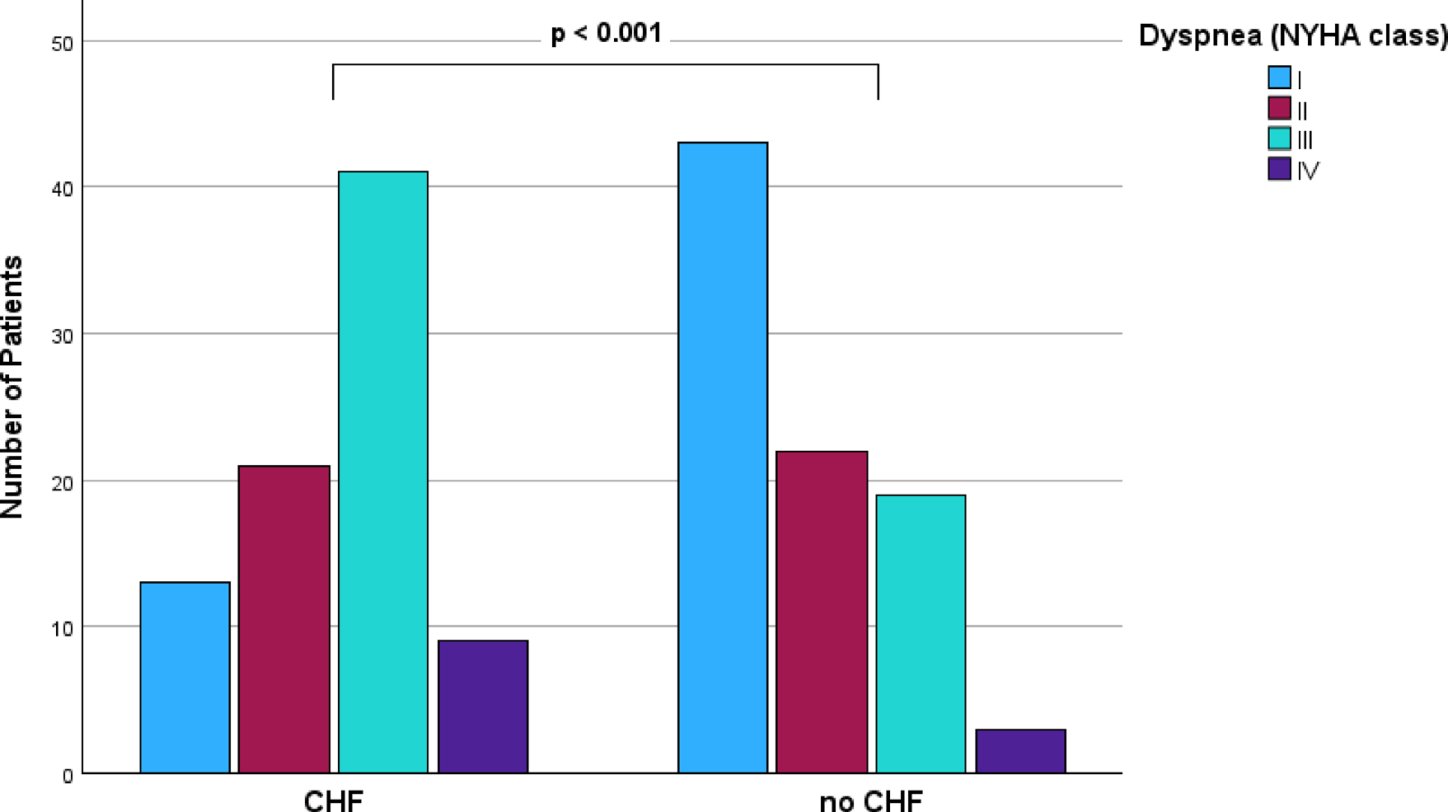
Dyspnea according to the NYHA-scale at admission for deferred patients (study group) with and without congestive heart failure (CHF, NT-proBNP > 900 pg/ml in combination with clinical symptoms) on the actual intervention date.

To further evaluate the clinical impact of CHF, deferred patients with CHF on the actual intervention date (NT-proBNP level > 900 pg/ml in combination with clinical symptoms; n = 89) were compared to those who were CHF-free at this time point (NT-proBNP level ≤ 900 pg/ml/no symptoms; n = 89). Clinical characteristics of both groups are displayed in Table 4. Median NT-proBNP level at admission was 2346 pg/ml (1512.5, 4330 pg/ml) in the group with and 228.5 pg/ml (111, 505 pg/ml) in the group without CHF. Patients with an NT-proBNP level > 900 pg/ml had higher NYHA classes (2.5 ± 0.9 vs. 1.8 ± 0.9; *p <*0.001, Fig. 2) and more impaired LVEF (2.2 ± 1.2 vs. 1.8 ± 1.0; *p <*0.001) when compared to those with an NT-proBNP level ≤ 900 pg/ml. Moreover, they had higher cTnT (30 (19, 52) vs. 11 (8, 19) ng/l; *p <*0.001) and creatinine levels (122.18 ± 78.24 vs. 91.08 ± 27.72 µmol/l; *p <*0.001) on the actual intervention date. Furthermore, patients with CHF were admitted more frequently as an emergency for their indented cardiac intervention (43.8% (39 patients) vs. 23.9% (21 patients); *p* = 0.005) and the interventional hospital stay lasted longer (3 (2, 8) vs. 2 (1, 3) days, *p <*0.001).

**Table 4:** Clinical characteristics of patients with and without congestive heart failure (NT-proBNP level > 900 pg/ml in combination with clinical symptoms) on the actual intervention date.

	NT-proBNP > 900 pg/ml + symptoms	NT-proBNP < = 900 pg/ml	*p* value
	n = 89	n = 89	
Parameters of cardiac status at admission			
NYHA class			
NYHA class average	2.54 ± 0.88	1.79 ± 0.90	** < 0.001**
NYHA I	13 (15.5)	43 (49.4)	
NYHA II	21 (25.0)	22 (25.0)	
NYHA III	41 (48.8)	19 (21.8)	
NYHA IV	9 (10.7)	3 (3.4)	
EHRA class	0.563 ± 1.10	0.72 ± 1.24	0.371
CCS class	0.77 ± 1.21	0.68 ± 1.27	0.634
cTnT (ng/l)	30 (19, 52)	11 (8, 19)	** < 0.001**
NT-proBNP (pg/ml)	2346.0 (1512.5, 4330.0)	228.50 (111.25, 505.25)	** < 0.001**
Creatinin (µmol/l)	122.18 ± 78.24	91.08 ± 27.72	** < 0.001**
LVEF grade	2.16 ± 1.17	1.82 ± 1.02	** < 0.001**
Data of hospital stay			
Hospital stay intervention (nights)	3 (2, 8)	2 (1, 3)	** < 0.001**
Emergency stay	39 (43.8)	21 (23.9)	**0.005**

NYHA, New York Heart Association; EHRA, European Heart Rhythm Association; CCS, Canadian Cardiovascular Society; cTnT, cardiac Troponin T; LVEF, left ventricular ejection fraction (grades: 1 normal, 2 mildly impaired, 3 moderately impaired, 4 severely impaired); significant differences are presented in bold.

**Fig. 3:**
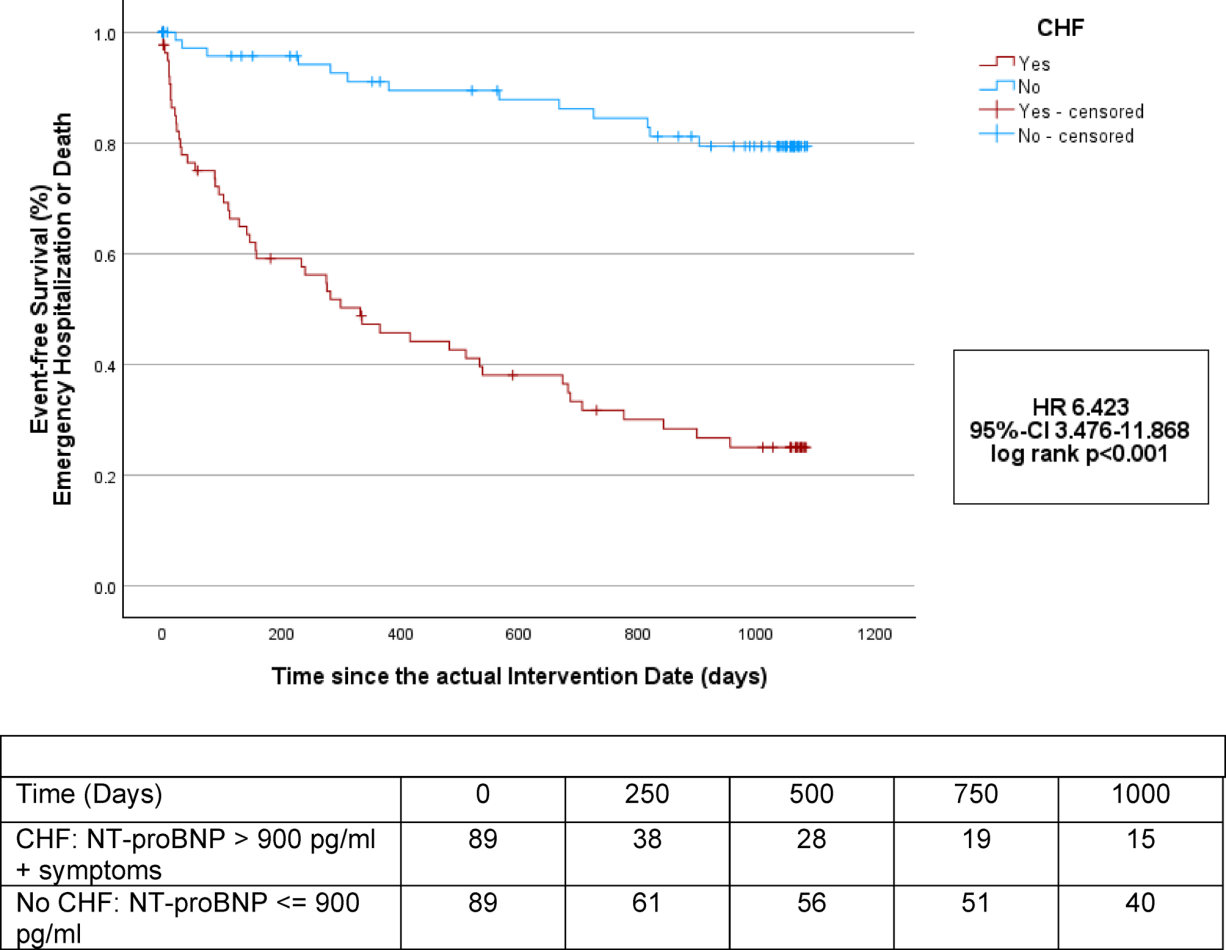
Kaplan–Meier estimators of the time to emergency cardiovascular hospitalization or death for deferred patients (study group) with and without congestive heart failure (CHF, NT-proBNP > 900 pg/ml in combination with clinical symptoms) starting from the actual, deferred intervention date.

Rates of emergency hospitalization or death over the postinterventional 36-month follow-up period were significantly higher and time-to-event was shorter in the group with CHF compared to the group without (57.3% vs. 14.8%, *p < *0.001; HR 6.423, 95%-CI 3.476–11.868, log rank *p < *0.001). Event rates were 42.7% (38 patients) vs. 6.8% (6 patients) at twelve months, 52.8% (47 patients) vs. 11.4% (10 patients) at 24 months and even 57.3% (51 patients) vs. 14.8% (13 patients) at 36 months after the procedure (each *p <*0.001). The first postinterventional event of emergency hospitalization and death occurred in patients with CHF after 334 (80.5, 365) days, and in patients without CHF after 365 (279.75, 365) days (*p* = 0.002). (Table 5). The corresponding Kaplan–Meier survival curves are shown in Fig. 3**.**

**Table 5:** Postinterventional clinical outcomes of patients with and without congestive heart failure (NT-proBNP level > 900 pg/ml in combination with clinical symptoms) on the actual intervention date.

	NT-proBNP > 900 pg/ml + symptoms	NT-proBNP < = 900 pg/ml	*p* value
	n = 89	n = 89	
Time-to-event			
Starting on the actual intervention date (days)	334 (80.5, 365)	365.00 (279.75, 365)	**0.002**
Postinterventional event rates			
MACE 12 months (%)	38 (42.7)	6 (6.8)	** < 0.001**
MACE 24 months (%)	47 (52.8)	10 (11.4)	** < 0.001**
MACE 36 months (%)	51 (57.3)	13 (14.8)	** < 0.001**

MACE, Major adverse cardiovascular events, defined as emergency cardiovascular hospitalization or death; significant differences are presented in bold.

### Subgroup analysis: deferred patients with planned transcatheter heart valve intervention

**Fig. 4:**
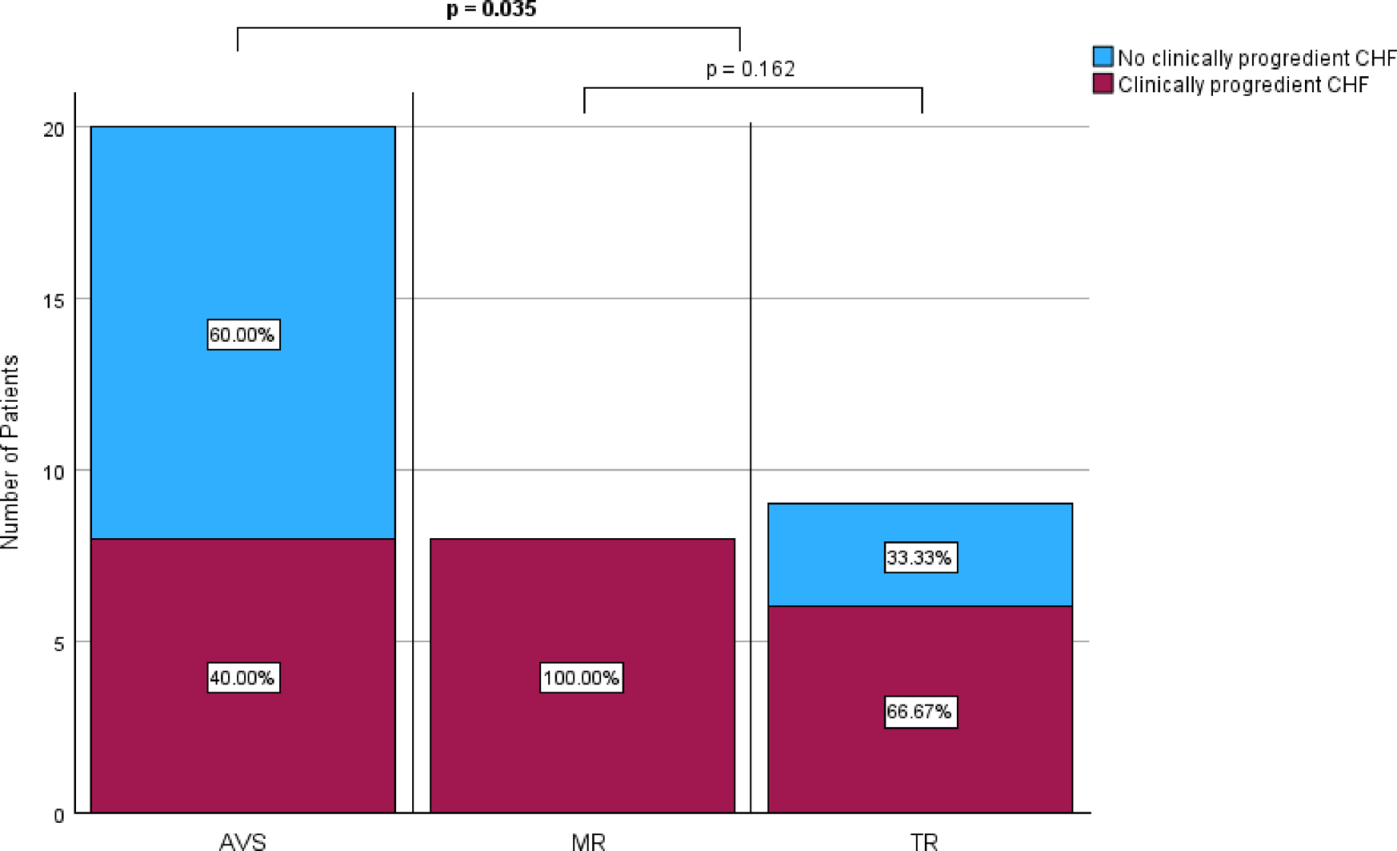
Proportion of study group patients with and without clinically progredient congestive heart failure (CHF, NT-proBNP > 900 pg/ml in combination with worsening of clinical symptoms) during the waiting time depending on the type of planned heart valve intervention.

In total, 49 deferred patients were scheduled for transcatheter heart valve intervention. Of these, 29 patients (59.2%) had aortic valve stenosis (AVS), 18 (36.7%) atrioventricular valve regurgitation, of which eight (16.3%) suffered from mitral valve regurgitation (MR) and ten (20.4%) from tricuspid valve regurgitation (TR), and two patients (4.2%) were scheduled for intervention of mitral valve stenosis (MS). Overall, 39 out of these 49 patients (79.6%) were diagnosed with CHF on the actual intervention date. In detail, 20 of 29 patients with AVS (69.0%), eight of eight patients with MR (100%) and nine of ten patients with TR (90.0%) had an NT-proBNP level of > 900 pg/ml. Of the patients for whom repeated data measurement were available, eighth of 20 patients with AVS (40.0%), eighth of eighth patients with MR (100%), and six of nine patients with TR (66.7%), had clinically progredient CHF during the waiting time. Risk was even higher in patients with planned intervention of the atrioventricular valves (mitral transcatheter edge-to-edge-repair (M-TEER) or tricuspid transcatheter edge-to-edge-repair (T-TEER)) compared to patients scheduled for transcatheter aortic valve replacement (TAVR) due to AVS (82.4% vs. 40.0%; *p* = 0.035 after Bonferroni correction). Between patients with planned intervention due to MR and TR, rates of CHF were not significantly different (*p* = 0.162 after Bonferroni correction) (Fig. 4).

### Impact of the predictor heart valve intervention on clinical outcomes in case of deferral

To further evaluate the effects of deferral in patients scheduled for transcatheter heart valve intervention, clinical outcomes were compared separately with those of regularly treated controls of the previous year 2019 (n = 47). No significant differences in baseline characteristics on the originally planned intervention date were observed between the subgroups of patients with planned heart valve intervention in the study and control group (Table 6). On the actual intervention date, deferred patients had significant higher NYHA-classes compared to the control group (2.9 ± 0.6 vs. 2.6 ± 0.7, *p* = 0.009) (supplementary Fig. 1). Furthermore, the planned transcatheter heart valve intervention had to be performed more frequently in the context of an emergency hospitalization (61.2% (30 patients) vs. 0% (0 patients), *p <*0.001). **(**Table 7**).** Emergency hospitalization or death occurred significantly more frequent during the 36 months after the originally planned intervention date in deferred patients compared to the regularly treated controls and time-to-event was shorter (83.7% vs. 40.4%, *p <*0.001; HR 4.37, 95%-CI 2.50–7.64, log rank *p <*0.001). Such an event occurred in 36 patients (73.5%) in the study group vs. seven patients (14.9%) in the control group after twelve months, in 39 (79.6%) vs. 13 patients (27.7%) after 24 months and in 41 (83.7%) vs. 19 patients (40.4%) after 36 months (each *p <*0.001). The first event of emergency hospitalization and death, starting from the originally planned treatment date, occurred in deferred patients of the study group after 38 (21, 311) and in the regularly treated patients of the control group after 366 (366, 366) days (*p <*0.001). (Table 8).

**Table 6:** Baseline characteristics of patients scheduled for transcatheter heart valve intervention: study group (deferred patients) and control group (regularly treated patients).

	Study group	Control group	*p* value
	n = 49	n = 47	
Age (years)	79.94 ± 6.56	79.47 ± 8.23	0.757
Male sex (%)	19 (38.8)	23 (48.9)	0.321
Height (cm)	173.90 ± 7.13	166.55 ± 9.43	0.060
Weight (kg)	83.20 ± 17.62	81.05 ± 21.32	0.805
Heart rate (bpm)	73 (65.5, 89)	60 (60.5, 80)	0.321
Blood pressure systolic (mmHg)	120.50 ± 14.54	121.75 ± 14.52	0.866
Blood pressure diastolic (mmHg)	68.38 ± 11.93	69.25 ± 10.29	0.877
Arterial hypertension (%)	22 (75.9)	36 (76.6)	0.943
Dyslipidemia (%)	9 (31.0)	23 (48.9)	0.123
Diabetes mellitus (%)	10 (34.5)	12 (25.5)	0.410
Familiy history (%)	1 (3.4)	6 (12.8)	0.125
Smoker (%)	6 (20.7)	7 (14.9)	0.521
Obesity (%)	6 (20.7)	11 (23.4)	0.786
History of TIA/stroke (%)	4 (13.8)	2 (4.3)	0.191
COPD (%)	3 (10.3)	4 (8.5)	0.792
OSAS (%)	2 (6.9)	2 (4.3)	0.622
Known CKD (%)	10 (34.5)	10 (21.3)	0.228
Known CAD (%)	20 (69.0)	31 (66.0)	0.790
Known cardiac arrhythmia (%)	18 (62.1)	23 (48.9)	0.271
NYHA class			
NYHA class average	2.42 ± 0.69	2.56 ± 0.69	0.908
NYHA I	2 (10.5)	3 (6.4)	
NYHA II	7 (36.8)	16 (35.6)	
NYHA III	10 (52.6)	24 (53.3)	
NYHA IV	0 (0)	2 (4.4)	
EHRA class	1.05 ± 0.23	0.07 ± 0.45	0.915
CCS class	0.53 ± 0.90	0.88 ± 1.03	0.187
cTnT (ng/l)	24 (18.75, 36.5)	19 (13, 30)	0.935
NT-proBNP (pg/ml)	1104 (449.75, 2270.75)	1534 (941, 4159)	0.217
Creatinin (µmol/l)	124.17 ± 62.51	102.87 ± 35.53	0.115
LVEF grade	2.15 ± 1.18	1.92 ± 1.20	0.699
LVEF normal	8 (40.0)	25 (54.3)	
LVEF mildly impaired	5 (25.0)	5 (10.9)	
LVEF moderately impaired	3 (15.0)	6 (13.0)	
LVEF severely impaired	4 (20.0)	10 (21.7)	

bpm, beats per minute; TIA, transient ischemic attack; COPD, chronic pulmonary obstructive disease; OSAS, obstructive sleep apnea syndrome; CKD, chronic kidney disease; CAD, coronary artery disease; NYHA, New York Heart Association; EHRA, European Heart Rhythm Association; CCS, Canadian Cardiovascular Society; LVEF, left ventricular ejection fraction (grades: 1 normal, 2 mildly impaired, 3 moderately impaired), 4 severely impaired); significant differences are presented in bold.

**Table 7:** Characteristics of patients scheduled for transcatheter heart valve intervention on the actual intervention date.

	Study group	Control group	*p* value
	n = 49	n = 47	
Parameters of cardiac status			
NYHA class			
NYHA class average	2.91 ± 0.58	2.56 ± 0.69	**0.009**
NYHA I	0 (0)	3 (6.4)	
NYHA II	10 (21.3)	16 (35.6)	
NYHA III	31 (66.0)	24 (53.3)	
NYHA IV	6 (12.8)	2 (4.4)	
EHRA class	0.12 ± 0.57	0.07 ± 0.45	0.624
CCS class	0.60 ± 1.12	0.88 ± 1.03	0.219
cTnT (ng/l)	27 (17, 42)	19 (13, 30)	0.051
NT-proBNP (pg/ml)	2347 (1095, 4278.5)	1534 (941, 4159)	0.186
Creatinin (µmol/l)	117.43 ± 56.73	102.87 ± 35.53	0.134
LVEF grade	1.81 ± 1.07	1.92 ± 1.20	0.407
Waiting time (days)	0 (0, 0)	26 (20.5, 41)	** < 0.001**
Data of hospital stay			
Hospital stay intervention (nights)	8 (6, 9.5)	6 (7, 9)	0.516
Emergency stay	30 (61.2)	0 (0, 0)	** < 0.001**

NYHA, New York Heart Association; EHRA, European Heart Rhythm Association; CCS, Canadian Cardiovascular Society; cTnT, cardiac Troponin T; LVEF, left ventricular ejection fraction (grades: 1 normal, 2 mildly impaired, 3 moderately impaired, 4 severely impaired); significant differences are presented in bold.

**Table 8:** Outcomes of patients scheduled for transcatheter heart valve intervention following the originally planned intervention date.

	Study group	Control group	*p* value
	n = 49	n = 47	
Time-to-event			
Starting on the originally planned intervention date (days)	38 (21, 311)	366 (366, 366)	** < 0.001**
Event rates following the originally planned intervention date			
MACE 12 months (%)	36 (73.5)	7 (14.9)	** < 0.001**
MACE 24 months (%)	39 (79.6)	13 (27.7)	** < 0.001**
MACE 36 months (%)	41 (83.7)	19 (40.4)	** < 0.001**

MACE, Major Adverse Cardiovascular Events, defined as emergency cardiovascular hospitalization or death; significant differences are presented in bold

**Fig. 5:**
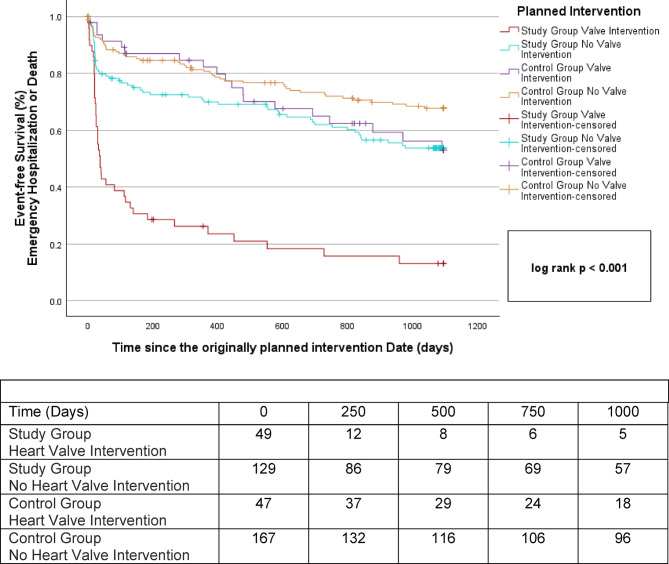
Kaplan–Meier estimators of the time to emergency cardiovascular hospitalization or death for deferred patients (study group) and regularly treated patients (control group) with and without planned heart valve intervention starting from the originally planned intervention date.

To substantiate the impact of the index disease on the outcome of emergency hospitalization or death following deferral, time-to-event of patients without the predictor heart valve interventions were also compared between the study and the control group. Notably, patients scheduled for other interventions, who were deferred and who were regularly treated had a comparable time-to-event during the 36-months follow-up period after the originally planned intervention date. Furthermore, while patients in the study group with deferred heart valve interventions had a shorter time-to-event then patients with other interventions, no such difference was observed in the control group. The corresponding Kaplan–Meier survival curves are also shown in Fig. 5.

## Discussion

In the present study, we analyzed criteria to identify high-risk patients for developing clinically progredient CHF during prolonged waiting time for scheduled non-emergency cardiac interventions. The main findings can be summarized as follows: About one-third of the deferred patients experienced clinically progredient CHF during the waiting period. The only independent predictor thereof was planned heart valve intervention, which was associated with a 35-fold increased risk of CHF with clinical progress during the waiting time. Patients scheduled for M/T-TEER were at higher risk than those with TAVR. CHF on the actual intervention date was associated with worse short- and long-term clinical outcomes, as evidenced by increased rates of emergency hospitalization and death over the following 36 months and a shorter time-to-event. Accordingly, increased event rates and a shorter time-to-event for deferred patients compared to the regularly treated controls following the originally planned intervention date were observed only in the subgroup with the predictor planned heart valve intervention.

While in our study the overall difference in CHF incidence between the deferred and regularly treated patients on the actual intervention date did not reach statistical significance (*p *= 0.078), our finding that one-third of the deferred patients experienced clinical progressive CHF indicates a considerable risk of CHF progression during the waiting time. Patients with heart failure prior to treatment showed worse short- and long-term outcomes, even post-procedure. This was evidenced by higher event rates and a more than six-fold shorter time to emergency hospitalization and death during the 36 month-follow-up period. Moreover, in patients with CHF, the deferred intervention had to be performed more often for reason of an emergency hospitalization and the hospital stay lasted longer. Finally, this goes along with an increased economic burden for the healthcare system. The drastic consequences of heart failure prior to treatment have been further highlighted in recent studies. A systematic review by Curio et al. reported that pre-existing heart failure with reduced ventricular function negatively influences outcomes after M-TEER^15–17^. Patients with advanced stage of disease as determined by NYHA class had worse outcomes after the intervention^16^. Furthermore, an analysis of the National Cardiovascular Data Registry (NCDR) of the American College of Cardiology (ACC) demonstrated that the presence of heart failure has been associated with a higher incidence of adverse events after percutaneous coronary intervention. Heart failure prior to intervention has been shown to independently predict 30-day mortality afterwards^18^.

NT-proBNP levels have been described as appropriate for the diagnosis of heart failure and are part of the definition in the guidelines^19^. It correlates with multiple effects of heart failure and can be used for diagnostic and prognostic approaches^20,21^. This was also shown by the getABI study, which demonstrated a significant correlation between NT-proBNP levels and both cardiovascular and all-cause mortality, indicating that NT-proBNP may serve as an independent risk predictor^22^. For patients with a mean age between 50 and 75 years, such as those in our cohort, a cut-off value of > 900 pg/ml has been recommended for the diagnosis of CHF^23^. In our study, in line with these data, an NT-proBNP level > 900 pg/ml was associated with significantly increased clinical signs of heart failure including advanced NYHA class, higher levels of the additional biomarkers cTnT and creatinine as well as worse LVEF grade. Moreover, in recent studies a comparable NT-proBNP cut-off was found to best predict poor clinical outcomes in deferred cardiac patients^10,24^. This underlines the suitability of this biomarker cut-off level to diagnose CHF, even in our study cohort. Nevertheless, patient characteristics, such as age and renal function, can impact NT-proBNP levels. To account for these factors, in accordance with current guidelines that incorporate clinical symptoms into the definition of CHF, elevated NT-proBNP levels were corroborated by the presence of heart failure symptoms. These symptoms included dyspnea, as assessed by NYHA class, emergency hospitalizations due to heart failure, and reduced LVEF, all of which are recognized clinical indicators of CHF^2^. Our observation of corresponding symptoms in all patients with elevated NT-proBNP levels supports the suitability of this biomarker as a screening tool for CHF in the present cohort of patients undergoing cardiovascular interventions.

Given the drastic consequences of CHF, it is essential to identify predictors associated with a high risk for clinically progredient CHF in the case of deferral to enable prevention. In the present analysis, we found that the criterion ‘scheduled transcatheter heart valve intervention’ independently predicted clinically progredient CHF during the prolonged waiting time. Patients with scheduled valve intervention exhibited a 35-fold increased risk of clinically progredient CHF. The comparability of relevant baseline characteristics, such as demographics, comorbidities, cardiac disease severity, and cardiac function, between deferred and regularly treated heart valve patients indicates that the poorer outcomes of deferred patients over 36 months are attributable to the deferral of treatment. Consistent with the unfavorable consequences of CHF, patients whose heart valve intervention was postponed had worse outcomes than those who received such intervention regularly, as evidenced by significantly higher rates of emergency hospitalization and death after twelve, 24, and 36 months post-procedure and a shorter time-to-event during the 36-month follow-up period. Higher NYHA functional class on admission indicated their poorer clinical status at the time of actual intervention. In addition, their intervention had to be performed more often in the context of an emergency hospitalization, which underlines the urgent need of performing heart valve interventions in time. In accordance with our findings, former studies have shown that timely heart valve intervention is essential to avoid unfavorable outcomes. Recent studies, such as the AS-DEFER study, investigating the effects of deferral of planned TAVR during the Covid-19 pandemic reported increased rates of disease-related hospitalizations due to symptoms or worsening of heart failure and mortality compared to regularly treated patients^25,26^. Adverse events occurred particularly during the waiting time, but even after the procedure performed.^10,25–27^. Cardiac event rates including accelerated symptoms and heart failure with need for urgent TAVR and death were high with 10% at one month and 35% at three months^28^. After two years, a mortality rate of even 50–68% was observed in untreated patients with symptomatic AVS, most of whom died of CHF^29,30^. Other reports describe mortality rates for untreated AVS at one, three, six, twelve and 24 months of 3.8, 10.4, 23.3, 27.5 and 41.1%^31,32^. Unfavorable consequences of postponement have also been observed in patients with deferral of intervention for atrioventricular valve regurgitation. A study of patients awaiting mitral valve repair reported a mortality rate of about 8% at one and a half months^33^. In patients scheduled for M-TEER at 180 days, a hospitalization rate for heart failure of about 50% and a mortality rate of about 10% have been reported^16^. In a large study of more than 10,000 patients with TR, an event rate of 40.1% for mortality or hospitalization for heart failure and an isolated mortality rate of 17.8% were observed at twelve months^34^. While these data suggest that AVS-patients are even more vulnerable, our results, in contrast, indicate an even higher risk of patients scheduled for intervention due to atrioventricular valve regurgitation, with rates of clinically progredient CHF at the end of the extended waiting time of over 80% for patients with planned M-/T-TEER compared to 40% for patients awaiting TAVR. However, these findings have to be interpreted with several confinements. In particular, we found an overall small and relatively higher number of studies of patients with AVS relative to studies of patients with atrioventricular valve regurgitation, which corresponds to the proportion of the single valve diseases in the present cohort. Furthermore, the broad variability of reported rates of emergency hospitalization for the reasons of valve-related complications or worsening of heart failure or death in AVS patients scheduled for aortic valve replacement (AVR), ranging from 10 to 19.6% within one month, suggests the presence of further influencing factors^26,28^. In particular, the proportion of patients actually treated, which was 100% in our study due to the study design, and the absolute waiting time may have an impact. In this term, in the above-mentioned study, the prognosis of TR patients may be negatively influenced by the low proportion of patients who underwent intervention of only 10%^34^. In conclusion, our data confirm, that all patients awaiting transcatheter heart valve intervention, TAVR or M-/T-TEER, are at very high risk of developing clinically progredient CHF during prolonged waiting time and the associated unfavorable clinical outcomes. However, our data particularly highlight the urgent need for timely treatment of patients with atrioventricular valve regurgitation, a condition that appears to be underappreciated in terms of its risks.

Despite the fact, that our analysis was conducted during the Covid-19 related lockdown, our results retain significant implications for contemporary management of cardiac disease. In the present study, a median postponement of the rather short period of 23 days was found to be associated with significantly worse outcomes in deferred patients with scheduled heart valve interventions. Even in absence of the Covid-19 pandemic patients often experience longer waiting times for scheduled cardiac procedures. Reports from the pre-Covid era found that patients with severe symptomatic AVS awaiting AVR experienced an average waiting time of about three months^27,35^. Due to the drastic rise of TAVR-procedures being performed, an increasing trend of waiting times was observed over time^35^. In a large Canadian study, the waiting time for TAVR was found to be twice that recommended by the guidelines for treatment of AVS^27^. In line with our results, recent studies reported that longer waiting times were associated with increasing mortality and hospitalizations related to heart failure as well as a reduction in quality-adjusted life years^35,36^. During the waiting time of 80 days a mortality rate of 4.3% was described^27^. However, beyond waiting times, changes in healthcare delivery during the Covid-19 lockdown may have influenced patients outcomes further. The pandemic and its associated restrictions caused significant disruptions in rehabilitation services, which play a crucial role in recovery for many conditions^37^. Medication adherence may have also declined due to reduced access to pharmacies and healthcare providers^38^. The widespread adoption of telemedicine and modifications to follow-up protocols altered the management of chronic diseases and continuity of care^39^. These systemic changes in healthcare delivery have been shown to impact long-term outcomes^40^. These circumstances may have particularly affected the most vulnerable patients, such as those with CHF or advanced valve disease, thereby intensifying the adverse effects of disease progression. Our data demonstrate impressively that even in patients with supposedly stable clinical condition transcatheter heart valve interventions and particularly interventions due to MR or TR should be conducted urgently and are not suitable for deferral, as this has been linked with poor outcomes.

## Conclusion

Given the severe consequences of CHF and the high incidence in patients with untreated cardiac disease, this study sought to identify patients at risk of developing clinically progredient CHF during prolonged waiting times for non-emergency cardiac interventions. Importantly, we found that patients with a planned heart valve intervention, such as TAVR, and especially M/T-TEER, are at very high risk of clinically progredient CHF during the prolonged waiting time, as measured by NT-proBNP levels in combination with worsening of clinical symptoms, including dyspnea as assessed by NYHA class, emergency cardiovascular hospitalization, and decline of LVEF. Moreover, our results underscore the serious implications of CHF, as the presence of this condition on the actual intervention date was associated with significantly worse short- and long-term clinical outcomes including increased rates of emergency cardiovascular hospitalization or death and a shorter time-to-event. Accordingly, clinical outcomes of patients with deferred transcatheter heart valve interventions are worse compared to regularly treated controls even after 36 months. Consequently, we want to emphasize the urgency of transcatheter heart valve interventions and in particular M-/T-TEER, which appears to be underappreciated, and mitigate against postponement of these interventions.

### Limitations

The results of our study have to be interpreted with several confinements. As this is a retrospective, observational, single center study it inherently has several limitations descriptive to this design. However, all consecutive patients in the defined time period were included without patient exclusion or preselection aiming to reduce selection bias as much as possible. Although the statistical adjustments enhance the robustness of the reported results within the present cohort, the monocentric design may limit generalizability of these results to other populations. Due to the variation in recommendations from the ESC and the American College of Cardiology (ACC)/ Society for Cardiovascular Angiography and Interventions (SCAI), regarding procedural prioritization strategies during the Covid-19 pandemic related lockdown^11,41^, procedural backlogs and institutional protocols can vary substantially across centers, particularly in the transatlantic setting. Additionally, due to the explorative character of the study, all results have to be interpreted as generating hypothesis. Furthermore, diagnosis of CHF primarily by laboratory analysis might be a subject for debate. However, measuring NT-proBNP levels is a strong criterion to diagnose CHF according to guideline recommendations, and this criterion was—also in line with the guidelines^2^—corroborated by clinical symptoms. Moreover, the cut-point was chosen in accordance to age-adjusted reference values, that have been confirmed by recent studies and have even proven to be suitable for the present collective.

## Supplementary Information

Below is the link to the electronic supplementary material.

Supplementary Material 1

## Data Availability

The data underlying this article will be shared on reasonable request to the corresponding author.
