# Supplementary material for: Deferral of scheduled transcatheter heart valve interventions strongly increases the risk of congestive heart failure
Source: Sci Rep. 2025 Sep 1;15:32151. doi: 10.1038/s41598-025-16742-7 (PMC12402251; doi:10.1038/s41598-025-16742-7)
Supplement: Supplementary file 1 — Supplementary Material 1 [file 41598_2025_16742_MOESM1_ESM.docx]

**Supplementary Fig. 1:** Dyspnea according to the NYHA-scale on the actual intervention date for patients with planned transcatheter heart valve intervention in the study group and control group.


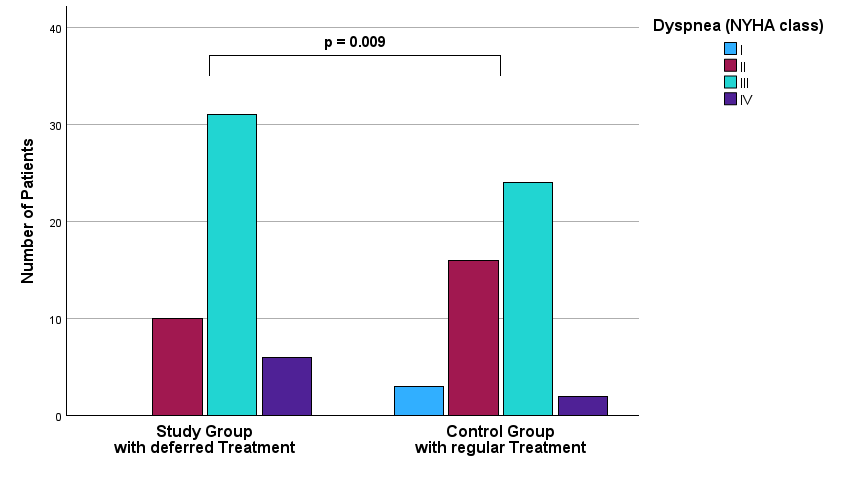


NYHA, New York Heart Association

**Supplementary Table 1: Univariate Binary Regression Analysis for Baseline Characteristics on the originally planned Intervention Date for Congestive Heart Failure (NT-proBNP level > 900 pg/ml in combination with clinical symptoms) with Clinical Progress during the Waiting Time (including all assessed Baseline Characteristics)**

|  | Odds ratio | 95%-Confidence interval | p-value |
| --- | --- | --- | --- |
| Age | 1.092 | 1.049 – 1.137 | **< 0.001** |
| Male Sex | 1.520 | 0.780 – 2.961 | 0.218 |
| Planned valve Intervention | 4.800 | 2.231 – 10.326 | **< 0.001** |
| Planned rhythmological procedure | 0.525 | 0.246 – 1.119 | 0.095 |
| Planned cardiac catheterization | 0.491 | 0.248 – 0.971 | **0.041** |
| Arterial hypertension | 1.496 | 0.607 – 3.691 | 0.382 |
| Hyperlipoproteinemia | 0.661 | 0.316 – 1.385 | 0.273 |
| Diabetes mellitus | 1.337 | 0.624 – 2.866 | 0.455 |
| Smoker | 0.697 | 0.326 – 1.491 | 0.352 |
| Positive family history | 0.899 | 0.317 – 2.549 | 0.842 |
| Obesitas | 1.540 | 0.661 – 3.589 | 0.317 |
| History of TIA or Stroke | 1.266 | 0.389 – 4.118 | 0.696 |
| COPD | 2.548 | 0.802 – 8.096 | 0.113 |
| OSAS | 1.000 | 0.238 – 4.198 | 1.000 |
| Known CAD | 1.588 | 0.646 – 3.902 | 0.314 |
| Known CKD | 2.641 | 1.089 – 6.404 | **0.032** |
| Known Cardiac Arrhythmia | 2.929 | 1.295 – 6.623 | **0.010** |
| Complains | 1.303 | 0.380 – 4.468 | 0.674 |
| NYHA class | 1.058 | 0.620 – 1.804 | 0.836 |
| EHRA class | 1.123 | 0.588 – 2.146 | 0.725 |
| CCS class | 0.865 | 0.579 – 1.294 | 0.481 |
| cTnT (ng/l) | 1.031 | 1.003 – 1.060 | **0.030** |
| NT-proBNP (pg/ml) | 1.000 | 1.000 – 1.000 | 0.121 |
| Creatinin (µmol/l) | 1.006 | 0.996 – 1.015 | 0.225 |
| LVEF (cat) |  |  | **0.024** |
| LVEF normal (ref) |  |  |  |
| LVEF mildly reduced | 2.022 | 0.554 – 7.387 | 0.287 |
| LVEF moderately impaired | 3.200 | 0.872 – 11.750 | 0.080 |
| LVEF severely reduced | 6.760 | 1.911 – 23.908 | **0.003** |
| TIA, transient ischemic attack; COPD, chronic pulmonary obstructive disease; OSAS, obstructive sleep apnea syndrome; CKD, chronic kidney disease; CAD, coronary artery disease; NYHA, New York Heart Association; EHRA, European Heart Rhythm Association; CCS, Canadian Cardiovascular Society; cTnT, cardiac Troponin T; LVEF, left ventricular ejection fraction (grades: 1 normal, 2 mildly impaired, 3 moderately impaired), 4 severely impaired); significant differences are presented in bold; | | | |
|  | | | |

**Supplementary Table 2:** **Pearson correlation analysis (r-value)**

|  | Planned Heart Valve Intervention | Age | LVEF severely reduced | Known cardiac arrhythmia | cTnT | Known CKD | Planned Cardiac Catheterization |
| --- | --- | --- | --- | --- | --- | --- | --- |
| Planned Heart Valve Intervention |  | 0.398 | -0.022 | 0.043 | 0.017 | 0.127 | -0.512 |
| Age |  |  | 0.069 | -0.023 | 0.317 | 0.072 | -0.078 |
| LVEF severely reduced |  |  |  | -0.047 | 0.394 | 0.159 | -0.002 |
| Known cardiac Arrhythmia |  |  |  |  | 0.033 | 0.175 | -0.456 |
| cTnT |  |  |  |  |  | 0.230 | 0.126 |
| Known CKD |  |  |  |  |  |  | -0.068 |
| Planned Cardiac Catheterization |  |  |  |  |  |  |  |

LVEF, left ventricular ejection fraction; cTnT, cardiac Troponin T; CKD, chronic kidney disease; high correlations r > I0.7I are presented in bold
